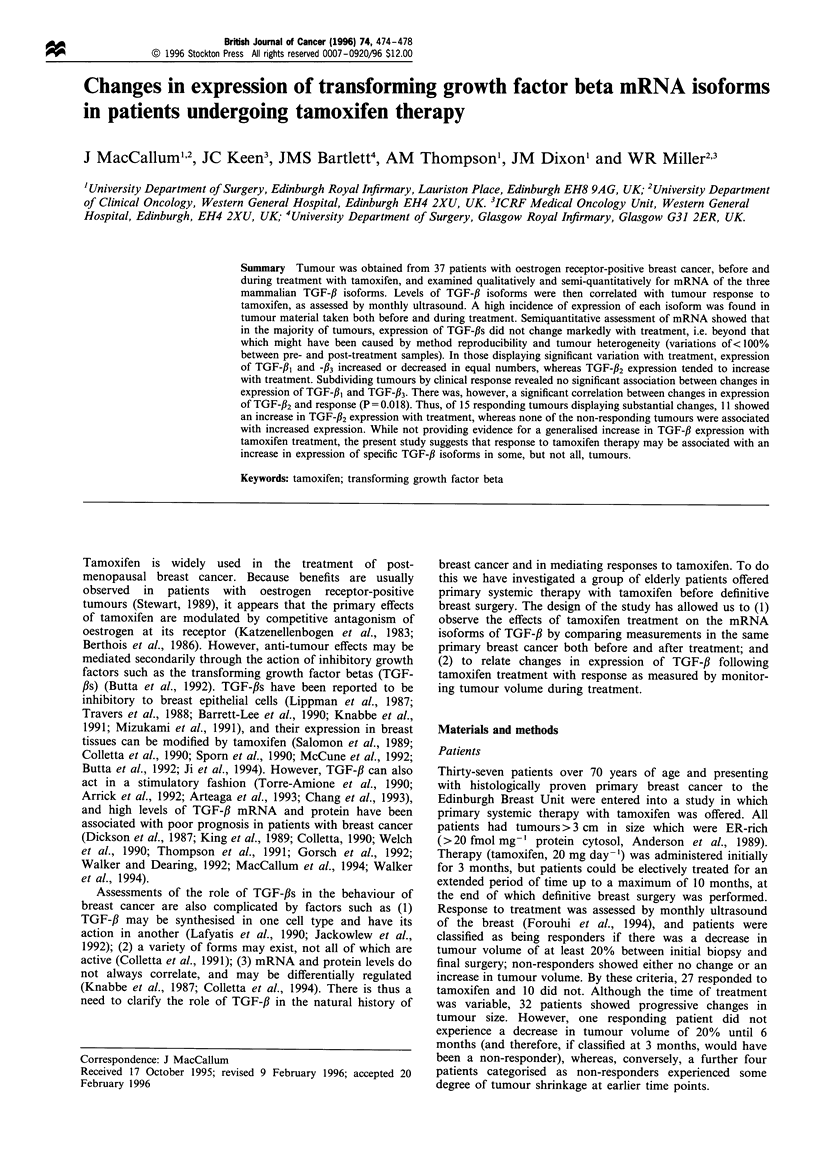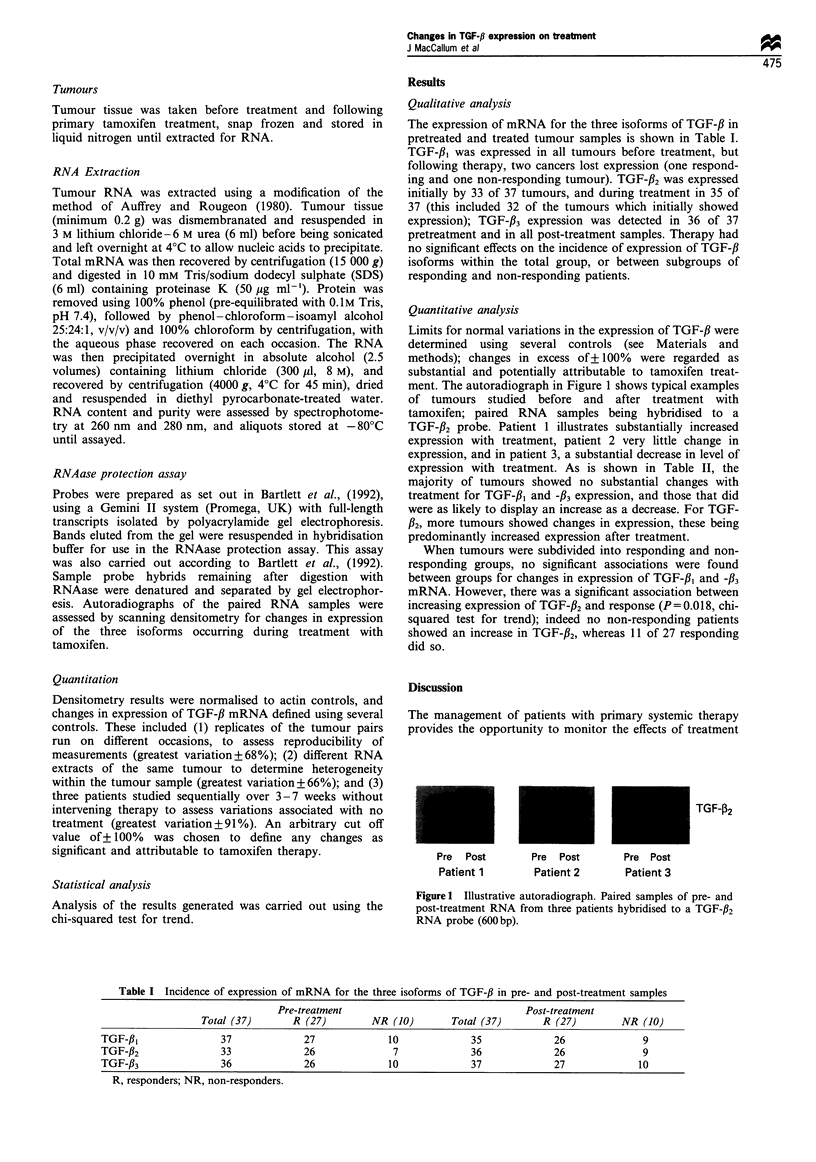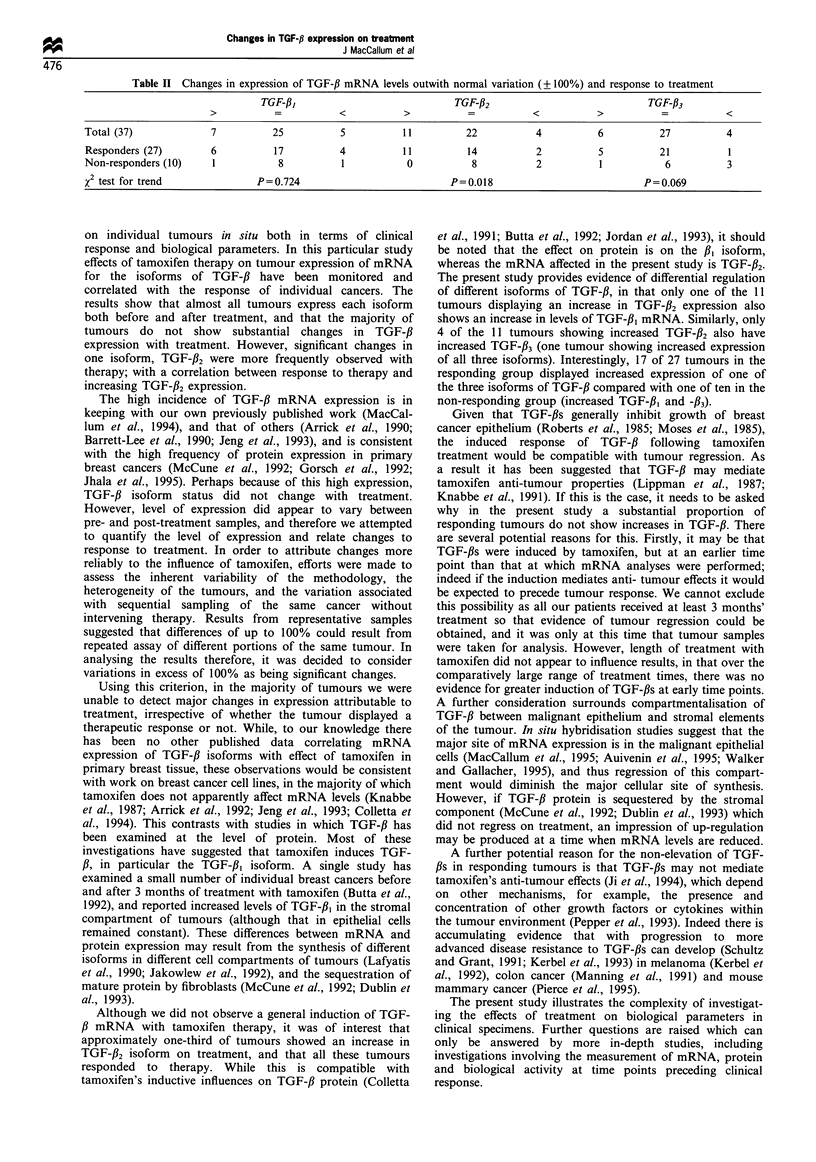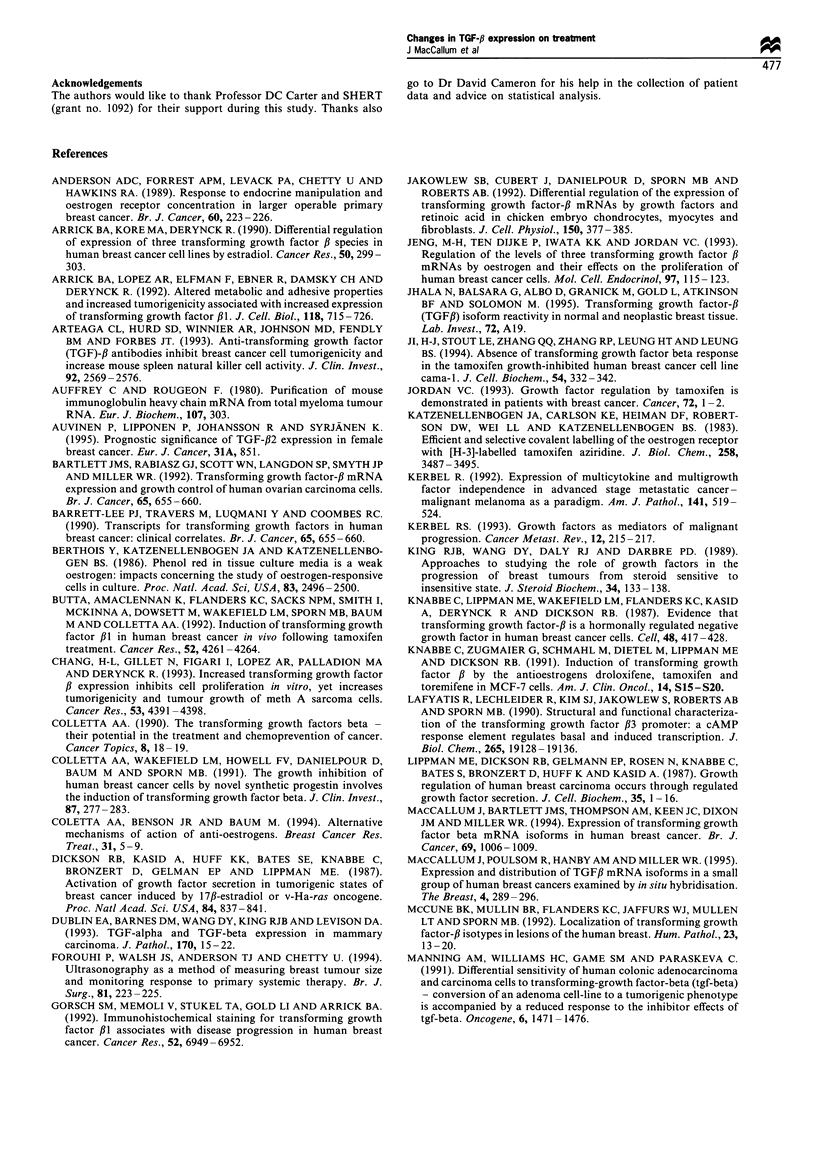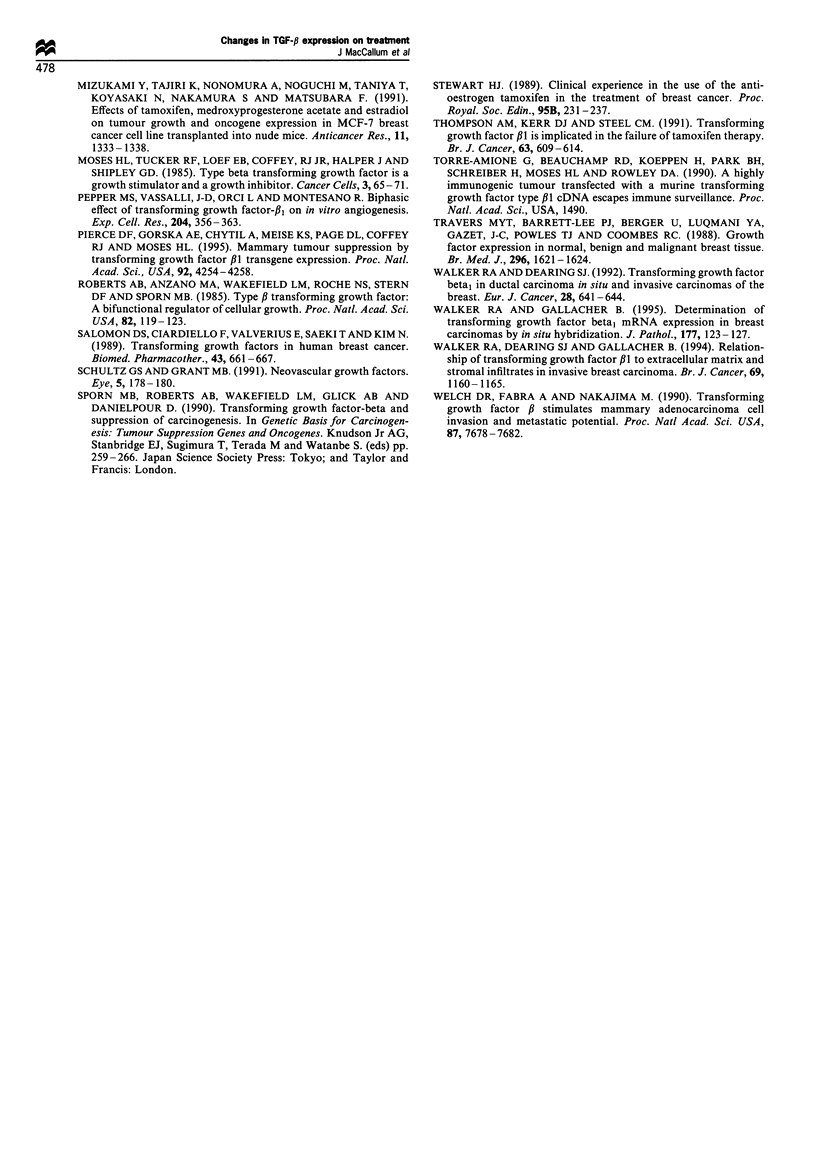# Changes in expression of transforming growth factor beta mRNA isoforms in patients undergoing tamoxifen therapy.

**DOI:** 10.1038/bjc.1996.385

**Published:** 1996-08

**Authors:** J. MacCallum, J. C. Keen, J. M. Bartlett, A. M. Thompson, J. M. Dixon, W. R. Miller

**Affiliations:** University Department of Surgery, Edinburgh Royal Infirmary, UK.

## Abstract

**Images:**


British Journal of Cancer (1996) 74, 474-478
© 1996 Stockton Press All rights reserved 0007-0920/96 $12.00

### Changes in expression of transforming growth factor beta mRNA isoforms in patients undergoing tamoxifen therapy

J MacCallum[1,2], JC Keen[3], JMS Bartlett[4], AM Thompson[1], JM Dixon[1] and WR Miller[2,3]

*[1]University Department of Surgery, Edinburgh Royal Infirmary, Lauriston Place, Edinburgh EH8 9AG, UK; [2]University Department of Clinical Oncology, Western General Hospital, Edinburgh EH4 2XU, UK. [3]ICRF Medical Oncology Unit, Western General Hospital, Edinburgh, EH4 2XU, UK; [4]University Department of Surgery, Glasgow Royal Infirmary, Glasgow G31 2ER, UK.*

**Summary** Tumour was obtained from 37 patients with oestrogen receptor-positive breast cancer, before and during treatment with tamoxifen, and examined qualitatively and semi-quantitatively for mRNA of the three mammalian TGF-$\beta$ isoforms. Levels of TGF-$\beta$ isoforms were then correlated with tumour response to tamoxifen, as assessed by monthly ultrasound. A high incidence of expression of each isoform was found in tumour material taken both before and during treatment. Semiquantitative assessment of mRNA showed that in the majority of tumours, expression of TGF-$\beta$s did not change markedly with treatment, i.e. beyond that which might have been caused by method reproducibility and tumour heterogeneity (variations of $<100\%$ between pre- and post-treatment samples). In those displaying significant variation with treatment, expression of TGF-$\beta_1$ and -$\beta_3$ increased or decreased in equal numbers, whereas TGF-$\beta_2$ expression tended to increase with treatment. Subdividing tumours by clinical response revealed no significant association between changes in expression of TGF-$\beta_1$ and TGF-$\beta_3$. There was, however, a significant correlation between changes in expression of TGF-$\beta_2$ and response ($P=0.018$). Thus, of 15 responding tumours displaying substantial changes, 11 showed an increase in TGF-$\beta_2$ expression with treatment, whereas none of the non-responding tumours were associated with increased expression. While not providing evidence for a generalised increase in TGF-$\beta$ expression with tamoxifen treatment, the present study suggests that response to tamoxifen therapy may be associated with an increase in expression of specific TGF-$\beta$ isoforms in some, but not all, tumours.

**Keywords:** tamoxifen; transforming growth factor beta

Tamoxifen is widely used in the treatment of postmenopausal breast cancer. Because benefits are usually observed in patients with oestrogen receptor-positive tumours (Stewart, 1989), it appears that the primary effects of tamoxifen are modulated by competitive antagonism of oestrogen at its receptor (Katzenellenbogen *et al.*, 1983; Berthois *et al.*, 1986). However, anti-tumour effects may be mediated secondarily through the action of inhibitory growth factors such as the transforming growth factor betas (TGF-$\beta$s) (Butta *et al.*, 1992). TGF-$\beta$s have been reported to be inhibitory to breast epithelial cells (Lippman *et al.*, 1987; Travers *et al.*, 1988; Barrett-Lee *et al.*, 1990; Knabbe *et al.*, 1991; Mizukami *et al.*, 1991), and their expression in breast tissues can be modified by tamoxifen (Salomon *et al.*, 1989; Colletta *et al.*, 1990; Sporn *et al.*, 1990; McCune *et al.*, 1992; Butta *et al.*, 1992; Ji *et al.*, 1994). However, TGF-$\beta$ can also act in a stimulatory fashion (Torre-Amione *et al.*, 1990; Arrick *et al.*, 1992; Arteaga *et al.*, 1993; Chang *et al.*, 1993), and high levels of TGF-$\beta$ mRNA and protein have been associated with poor prognosis in patients with breast cancer (Dickson *et al.*, 1987; King *et al.*, 1989; Colletta, 1990; Welch *et al.*, 1990; Thompson *et al.*, 1991; Gorsch *et al.*, 1992; Walker and Dearing, 1992; MacCallum *et al.*, 1994; Walker *et al.*, 1994).

Assessments of the role of TGF-$\beta$s in the behaviour of breast cancer are also complicated by factors such as (1) TGF-$\beta$ may be synthesised in one cell type and have its action in another (Lafyatis *et al.*, 1990; Jackowlew *et al.*, 1992); (2) a variety of forms may exist, not all of which are active (Colletta *et al.*, 1991); (3) mRNA and protein levels do not always correlate, and may be differentially regulated (Knabbe *et al.*, 1987; Colletta *et al.*, 1994). There is thus a need to clarify the role of TGF-$\beta$ in the natural history of breast cancer and in mediating responses to tamoxifen. To do this we have investigated a group of elderly patients offered primary systemic therapy with tamoxifen before definitive breast surgery. The design of the study has allowed us to (1) observe the effects of tamoxifen treatment on the mRNA isoforms of TGF-$\beta$ by comparing measurements in the same primary breast cancer both before and after treatment; and (2) to relate changes in expression of TGF-$\beta$ following tamoxifen treatment with response as measured by monitoring tumour volume during treatment.

Correspondence: J MacCallum
Received 17 October 1995; revised 9 February 1996; accepted 20 February 1996

### Materials and methods

### *Patients*

Thirty-seven patients over 70 years of age and presenting with histologically proven primary breast cancer to the Edinburgh Breast Unit were entered into a study in which primary systemic therapy with tamoxifen was offered. All patients had tumours $>3$ cm in size which were ER-rich ($>20$ fmol mg$^{-1}$ protein cytosol, Anderson *et al.*, 1989). Therapy (tamoxifen, 20 mg day$^{-1}$) was administered initially for 3 months, but patients could be electively treated for an extended period of time up to a maximum of 10 months, at the end of which definitive breast surgery was performed. Response to treatment was assessed by monthly ultrasound of the breast (Forouhi *et al.*, 1994), and patients were classified as being responders if there was a decrease in tumour volume of at least 20% between initial biopsy and final surgery; non-responders showed either no change or an increase in tumour volume. By these criteria, 27 responded to tamoxifen and 10 did not. Although the time of treatment was variable, 32 patients showed progressive changes in tumour size. However, one responding patient did not experience a decrease in tumour volume of 20% until 6 months (and therefore, if classified at 3 months, would have been a non-responder), whereas, conversely, a further four patients categorised as non-responders experienced some degree of tumour shrinkage at earlier time points.

*Tumours*

Tumour tissue was taken before treatment and following primary tamoxifen treatment, snap frozen and stored in liquid nitrogen until extracted for RNA.

*RNA Extraction*

Tumour RNA was extracted using a modification of the method of Auffrey and Rougeon (1980). Tumour tissue (minimum 0.2 g) was dismembranated and resuspended in 3 M lithium chloride–6 M urea (6 ml) before being sonicated and left overnight at 4°C to allow nucleic acids to precipitate. Total mRNA was then recovered by centrifugation (15 000 *g*) and digested in 10 mM Tris/sodium dodecyl sulphate (SDS) (6 ml) containing proteinase K (50 $\mu$g ml$^{-1}$). Protein was removed using 100% phenol (pre-equilibrated with 0.1M Tris, pH 7.4), followed by phenol–chloroform–isoamyl alcohol 25:24:1, v/v/v) and 100% chloroform by centrifugation, with the aqueous phase recovered on each occasion. The RNA was then precipitated overnight in absolute alcohol (2.5 volumes) containing lithium chloride (300 $\mu$l, 8 M), and recovered by centrifugation (4000 *g*, 4°C for 45 min), dried and resuspended in diethyl pyrocarbonate-treated water. RNA content and purity were assessed by spectrophotometry at 260 nm and 280 nm, and aliquots stored at −80°C until assayed.

*RNAase protection assay*

Probes were prepared as set out in Bartlett *et al.*, (1992), using a Gemini II system (Promega, UK) with full-length transcripts isolated by polyacrylamide gel electrophoresis. Bands eluted from the gel were resuspended in hybridisation buffer for use in the RNAase protection assay. This assay was also carried out according to Bartlett *et al.*, (1992). Sample probe hybrids remaining after digestion with RNAase were denatured and separated by gel electrophoresis. Autoradiographs of the paired RNA samples were assessed by scanning densitometry for changes in expression of the three isoforms occurring during treatment with tamoxifen.

*Quantitation*

Densitometry results were normalised to actin controls, and changes in expression of TGF-$\beta$ mRNA defined using several controls. These included (1) replicates of the tumour pairs run on different occasions, to assess reproducibility of measurements (greatest variation ± 68%); (2) different RNA extracts of the same tumour to determine heterogeneity within the tumour sample (greatest variation ± 66%); and (3) three patients studied sequentially over 3–7 weeks without intervening therapy to assess variations associated with no treatment (greatest variation ± 91%). An arbitrary cut off value of ± 100% was chosen to define any changes as significant and attributable to tamoxifen therapy.

*Statistical analysis*

Analysis of the results generated was carried out using the chi-squared test for trend.

### Results

*Qualitative analysis*

The expression of mRNA for the three isoforms of TGF-$\beta$ in pretreated and treated tumour samples is shown in Table I. TGF-$\beta_1$ was expressed in all tumours before treatment, but following therapy, two cancers lost expression (one responding and one non-responding tumour). TGF-$\beta_2$ was expressed initially by 33 of 37 tumours, and during treatment in 35 of 37 (this included 32 of the tumours which initially showed expression); TGF-$\beta_3$ expression was detected in 36 of 37 pretreatment and in all post-treatment samples. Therapy had no significant effects on the incidence of expression of TGF-$\beta$ isoforms within the total group, or between subgroups of responding and non-responding patients.

*Quantitative analysis*

Limits for normal variations in the expression of TGF-$\beta$ were determined using several controls (see Materials and methods); changes in excess of ± 100% were regarded as substantial and potentially attributable to tamoxifen treatment. The autoradiograph in Figure 1 shows typical examples of tumours studied before and after treatment with tamoxifen; paired RNA samples being hybridised to a TGF-$\beta_2$ probe. Patient 1 illustrates substantially increased expression with treatment, patient 2 very little change in expression, and in patient 3, a substantial decrease in level of expression with treatment. As is shown in Table II, the majority of tumours showed no substantial changes with treatment for TGF-$\beta_1$ and -$\beta_3$ expression, and those that did were as likely to display an increase as a decrease. For TGF-$\beta_2$, more tumours showed changes in expression, these being predominantly increased expression after treatment.

When tumours were subdivided into responding and non-responding groups, no significant associations were found between groups for changes in expression of TGF-$\beta_1$ and -$\beta_3$ mRNA. However, there was a significant association between increasing expression of TGF-$\beta_2$ and response ($P = 0.018$, chi-squared test for trend); indeed no non-responding patients showed an increase in TGF-$\beta_2$, whereas 11 of 27 responding did so.

### Discussion

The management of patients with primary systemic therapy provides the opportunity to monitor the effects of treatment

**Figure 1** Illustrative autoradiograph. Paired samples of pre- and post-treatment RNA from three patients hybridised to a TGF-$\beta_2$ RNA probe (600 bp).

**Table I** Incidence of expression of mRNA for the three isoforms of TGF-$\beta$ in pre- and post-treatment samples

| | | *Pre-treatment* | | | *Post-treatment* | |
|---|---|---|---|---|---|---|
| | *Total (37)* | *R (27)* | *NR (10)* | *Total (37)* | *R (27)* | *NR (10)* |
| TGF-$\beta_1$ | 37 | 27 | 10 | 35 | 26 | 9 |
| TGF-$\beta_2$ | 33 | 26 | 7 | 36 | 26 | 9 |
| TGF-$\beta_3$ | 36 | 26 | 10 | 37 | 27 | 10 |

R, responders; NR, non-responders.

**Table II** Changes in expression of TGF-$\beta$ mRNA levels outwith normal variation ($\pm$100%) and response to treatment

| | TGF-$\beta_1$ | | | TGF-$\beta_2$ | | | TGF-$\beta_3$ | | |
|---|---|---|---|---|---|---|---|---|---|
| | > | = | < | > | = | < | > | = | < |
| Total (37) | 7 | 25 | 5 | 11 | 22 | 4 | 6 | 27 | 4 |
| Responders (27) | 6 | 17 | 4 | 11 | 14 | 2 | 5 | 21 | 1 |
| Non-responders (10) | 1 | 8 | 1 | 0 | 8 | 2 | 1 | 6 | 3 |
| $\chi^2$ test for trend | | $P=0.724$ | | | $P=0.018$ | | | $P=0.069$ | |

on individual tumours *in situ* both in terms of clinical response and biological parameters. In this particular study effects of tamoxifen therapy on tumour expression of mRNA for the isoforms of TGF-$\beta$ have been monitored and correlated with the response of individual cancers. The results show that almost all tumours express each isoform both before and after treatment, and that the majority of tumours do not show substantial changes in TGF-$\beta$ expression with treatment. However, significant changes in one isoform, TGF-$\beta_2$ were more frequently observed with therapy; with a correlation between response to therapy and increasing TGF-$\beta_2$ expression.

The high incidence of TGF-$\beta$ mRNA expression is in keeping with our own previously published work (MacCallum *et al.*, 1994), and that of others (Arrick *et al.*, 1990; Barrett-Lee *et al.*, 1990; Jeng *et al.*, 1993), and is consistent with the high frequency of protein expression in primary breast cancers (McCune *et al.*, 1992; Gorsch *et al.*, 1992; Jhala *et al.*, 1995). Perhaps because of this high expression, TGF-$\beta$ isoform status did not change with treatment. However, level of expression did appear to vary between pre- and post-treatment samples, and therefore we attempted to quantify the level of expression and relate changes to response to treatment. In order to attribute changes more reliably to the influence of tamoxifen, efforts were made to assess the inherent variability of the methodology, the heterogeneity of the tumours, and the variation associated with sequential sampling of the same cancer without intervening therapy. Results from representative samples suggested that differences of up to 100% could result from repeated assay of different portions of the same tumour. In analysing the results therefore, it was decided to consider variations in excess of 100% as being significant changes.

Using this criterion, in the majority of tumours we were unable to detect major changes in expression attributable to treatment, irrespective of whether the tumour displayed a therapeutic response or not. While, to our knowledge there has been no other published data correlating mRNA expression of TGF-$\beta$ isoforms with effect of tamoxifen in primary breast tissue, these observations would be consistent with work on breast cancer cell lines, in the majority of which tamoxifen does not apparently affect mRNA levels (Knabbe *et al.*, 1987; Arrick *et al.*, 1992; Jeng *et al.*, 1993; Colletta *et al.*, 1994). This contrasts with studies in which TGF-$\beta$ has been examined at the level of protein. Most of these investigations have suggested that tamoxifen induces TGF-$\beta$, in particular the TGF-$\beta_1$ isoform. A single study has examined a small number of individual breast cancers before and after 3 months of treatment with tamoxifen (Butta *et al.*, 1992), and reported increased levels of TGF-$\beta_1$ in the stromal compartment of tumours (although that in epithelial cells remained constant). These differences between mRNA and protein expression may result from the synthesis of different isoforms in different cell compartments of tumours (Lafyatis *et al.*, 1990; Jakowlew *et al.*, 1992), and the sequestration of mature protein by fibroblasts (McCune *et al.*, 1992; Dublin *et al.*, 1993).

Although we did not observe a general induction of TGF-$\beta$ mRNA with tamoxifen therapy, it was of interest that approximately one-third of tumours showed an increase in TGF-$\beta_2$ isoform on treatment, and that all these tumours responded to therapy. While this is compatible with tamoxifen's inductive influences on TGF-$\beta$ protein (Colletta *et al.*, 1991; Butta *et al.*, 1992; Jordan *et al.*, 1993), it should be noted that the effect on protein is on the $\beta_1$ isoform, whereas the mRNA affected in the present study is TGF-$\beta_2$. The present study provides evidence of differential regulation of different isoforms of TGF-$\beta$, in that only one of the 11 tumours displaying an increase in TGF-$\beta_2$ expression also shows an increase in levels of TGF-$\beta_1$ mRNA. Similarly, only 4 of the 11 tumours showing increased TGF-$\beta_2$ also have increased TGF-$\beta_3$ (one tumour showing increased expression of all three isoforms). Interestingly, 17 of 27 tumours in the responding group displayed increased expression of one of the three isoforms of TGF-$\beta$ compared with one of ten in the non-responding group (increased TGF-$\beta_1$ and -$\beta_3$).

Given that TGF-$\beta$s generally inhibit growth of breast cancer epithelium (Roberts *et al.*, 1985; Moses *et al.*, 1985), the induced response of TGF-$\beta$ following tamoxifen treatment would be compatible with tumour regression. As a result it has been suggested that TGF-$\beta$ may mediate tamoxifen anti-tumour properties (Lippman *et al.*, 1987; Knabbe *et al.*, 1991). If this is the case, it needs to be asked why in the present study a substantial proportion of responding tumours do not show increases in TGF-$\beta$. There are several potential reasons for this. Firstly, it may be that TGF-$\beta$s were induced by tamoxifen, but at an earlier time point than that at which mRNA analyses were performed; indeed if the induction mediates anti- tumour effects it would be expected to precede tumour response. We cannot exclude this possibility as all our patients received at least 3 months' treatment so that evidence of tumour regression could be obtained, and it was only at this time that tumour samples were taken for analysis. However, length of treatment with tamoxifen did not appear to influence results, in that over the comparatively large range of treatment times, there was no evidence for greater induction of TGF-$\beta$s at early time points. A further consideration surrounds compartmentalisation of TGF-$\beta$ between malignant epithelium and stromal elements of the tumour. *In situ* hybridisation studies suggest that the major site of mRNA expression is in the malignant epithelial cells (MacCallum *et al.*, 1995; Auivenin *et al.*, 1995; Walker and Gallacher, 1995), and thus regression of this compartment would diminish the major cellular site of synthesis. However, if TGF-$\beta$ protein is sequestered by the stromal component (McCune *et al.*, 1992; Dublin *et al.*, 1993) which did not regress on treatment, an impression of up-regulation may be produced at a time when mRNA levels are reduced.

A further potential reason for the non-elevation of TGF-$\beta$s in responding tumours is that TGF-$\beta$s may not mediate tamoxifen's anti-tumour effects (Ji *et al.*, 1994), which depend on other mechanisms, for example, the presence and concentration of other growth factors or cytokines within the tumour environment (Pepper *et al.*, 1993). Indeed there is accumulating evidence that with progression to more advanced disease resistance to TGF-$\beta$s can develop (Schultz and Grant, 1991; Kerbel *et al.*, 1993) in melanoma (Kerbel *et al.*, 1992), colon cancer (Manning *et al.*, 1991) and mouse mammary cancer (Pierce *et al.*, 1995).

The present study illustrates the complexity of investigating the effects of treatment on biological parameters in clinical specimens. Further questions are raised which can only be answered by more in-depth studies, including investigations involving the measurement of mRNA, protein and biological activity at time points preceding clinical response.

**Acknowledgements**
The authors would like to thank Professor DC Carter and SHERT (grant no. 1092) for their support during this study. Thanks also go to Dr David Cameron for his help in the collection of patient data and advice on statistical analysis.

### References

ANDERSON ADC, FORREST APM, LEVACK PA, CHETTY U AND HAWKINS RA. (1989). Response to endocrine manipulation and oestrogen receptor concentration in larger operable primary breast cancer. *Br. J. Cancer*, **60**, 223–226.

ARRICK BA, KORE MA, DERYNCK R. (1990). Differential regulation of expression of three transforming growth factor β species in human breast cancer cell lines by estradiol. *Cancer Res.*, **50**, 299–303.

ARRICK BA, LOPEZ AR, ELFMAN F, EBNER R, DAMSKY CH AND DERYNCK R. (1992). Altered metabolic and adhesive properties and increased tumorigenicity associated with increased expression of transforming growth factor β1. *J. Cell. Biol.*, **118**, 715–726.

ARTEAGA CL, HURD SD, WINNIER AR, JOHNSON MD, FENDLY BM AND FORBES JT. (1993). Anti-transforming growth factor (TGF)-β antibodies inhibit breast cancer cell tumorigenicity and increase mouse spleen natural killer cell activity. *J. Clin. Invest.*, **92**, 2569–2576.

AUFFREY C AND ROUGEON F. (1980). Purification of mouse immunoglobulin heavy chain mRNA from total myeloma tumour RNA. *Eur. J. Biochem.*, **107**, 303.

AUVINEN P, LIPPONEN P, JOHANSSON R AND SYRJÄNEN K. (1995). Prognostic significance of TGF-β2 expression in female breast cancer. *Eur. J. Cancer*, **31A**, 851.

BARTLETT JMS, RABIASZ GJ, SCOTT WN, LANGDON SP, SMYTH JP AND MILLER WR. (1992). Transforming growth factor-β mRNA expression and growth control of human ovarian carcinoma cells. *Br. J. Cancer*, **65**, 655–660.

BARRETT-LEE PJ, TRAVERS M, LUQMANI Y AND COOMBES RC. (1990). Transcripts for transforming growth factors in human breast cancer: clinical correlates. *Br. J. Cancer*, **65**, 655–660.

BERTHOIS Y, KATZENELLENBOGEN JA AND KATZENELLENBOGEN BS. (1986). Phenol red in tissue culture media is a weak oestrogen: impacts concerning the study of oestrogen-responsive cells in culture. *Proc. Natl. Acad. Sci, USA*, **83**, 2496–2500.

BUTTA, AMACLENNAN K, FLANDERS KC, SACKS NPM, SMITH I, MCKINNA A, DOWSETT M, WAKEFIELD LM, SPORN MB, BAUM M AND COLLETTA AA. (1992). Induction of transforming growth factor β1 in human breast cancer *in vivo* following tamoxifen treatment. *Cancer Res.*, **52**, 4261–4264.

CHANG, H-L, GILLET N, FIGARI I, LOPEZ AR, PALLADION MA AND DERYNCK R. (1993). Increased transforming growth factor β expression inhibits cell proliferation *in vitro*, yet increases tumorigenicity and tumour growth of meth A sarcoma cells. *Cancer Res.*, **53**, 4391–4398.

COLLETTA AA. (1990). The transforming growth factors beta – their potential in the treatment and chemoprevention of cancer. *Cancer Topics*, **8**, 18–19.

COLLETTA AA, WAKEFIELD LM, HOWELL FV, DANIELPOUR D, BAUM M AND SPORN MB. (1991). The growth inhibition of human breast cancer cells by novel synthetic progestin involves the induction of transforming growth factor beta. *J. Clin. Invest.*, **87**, 277–283.

COLETTA AA, BENSON JR AND BAUM M. (1994). Alternative mechanisms of action of anti-oestrogens. *Breast Cancer Res. Treat.*, **31**, 5–9.

DICKSON RB, KASID A, HUFF KK, BATES SE, KNABBE C, BRONZERT D, GELMAN EP AND LIPPMAN ME. (1987). Activation of growth factor secretion in tumorigenic states of breast cancer induced by 17β-estradiol or v-Ha-*ras* oncogene. *Proc. Natl Acad. Sci. USA*, **84**, 837–841.

DUBLIN EA, BARNES DM, WANG DY, KING RJB AND LEVISON DA. (1993). TGF-alpha and TGF-beta expression in mammary carcinoma. *J. Pathol.*, **170**, 15–22.

FOROUHI P, WALSH JS, ANDERSON TJ AND CHETTY U. (1994). Ultrasonography as a method of measuring breast tumour size and monitoring response to primary systemic therapy. *Br. J. Surg.*, **81**, 223–225.

GORSCH SM, MEMOLI V, STUKEL TA, GOLD LI AND ARRICK BA. (1992). Immunohistochemical staining for transforming growth factor β1 associates with disease progression in human breast cancer. *Cancer Res.*, **52**, 6949–6952.

JAKOWLEW SB, CUBERT J, DANIELPOUR D, SPORN MB AND ROBERTS AB. (1992). Differential regulation of the expression of transforming growth factor-β mRNAs by growth factors and retinoic acid in chicken embryo chondrocytes, myocytes and fibroblasts. *J. Cell. Physiol.*, **150**, 377–385.

JENG, M-H, TEN DIJKE P, IWATA KK AND JORDAN VC. (1993). Regulation of the levels of three transforming growth factor β mRNAs by oestrogen and their effects on the proliferation of human breast cancer cells. *Mol. Cell. Endocrinol*, **97**, 115–123.

JHALA N, BALSARA G, ALBO D, GRANICK M, GOLD L, ATKINSON BF AND SOLOMON M. (1995). Transforming growth factor-β (TGFβ) isoform reactivity in normal and neoplastic breast tissue. *Lab. Invest.*, **72**, A19.

JI, H-J, STOUT LE, ZHANG QQ, ZHANG RP, LEUNG HT AND LEUNG BS. (1994). Absence of transforming growth factor beta response in the tamoxifen growth-inhibited human breast cancer cell line cama-1. *J. Cell. Biochem.*, **54**, 332–342.

JORDAN VC. (1993). Growth factor regulation by tamoxifen is demonstrated in patients with breast cancer. *Cancer*, **72**, 1–2.

KATZENELLENBOGEN JA, CARLSON KE, HEIMAN DF, ROBERTSON DW, WEI LL AND KATZENELLENBOGEN BS. (1983). Efficient and selective covalent labelling of the oestrogen receptor with [H-3]-labelled tamoxifen aziridine. *J. Biol. Chem.*, **258**, 3487–3495.

KERBEL R. (1992). Expression of multicytokine and multigrowth factor independence in advanced stage metastatic cancer–malignant melanoma as a paradigm. *Am. J. Pathol.*, **141**, 519–524.

KERBEL RS. (1993). Growth factors as mediators of malignant progression. *Cancer Metast. Rev.*, **12**, 215–217.

KING RJB, WANG DY, DALY RJ AND DARBRE PD. (1989). Approaches to studying the role of growth factors in the progression of breast tumours from steroid sensitive to insensitive state. *J. Steroid Biochem.*, **34**, 133–138.

KNABBE C, LIPPMAN ME, WAKEFIELD LM, FLANDERS KC, KASID A, DERYNCK R AND DICKSON RB. (1987). Evidence that transforming growth factor-β is a hormonally regulated negative growth factor in human breast cancer cells. *Cell*, **48**, 417–428.

KNABBE C, ZUGMAIER G, SCHMAHL M, DIETEL M, LIPPMAN ME AND DICKSON RB. (1991). Induction of transforming growth factor β by the antioestrogens droloxifene, tamoxifen and toremifene in MCF-7 cells. *Am. J. Clin. Oncol.*, **14**, **S15–S20.**

LAFYATIS R, LECHLEIDER R, KIM SJ, JAKOWLEW S, ROBERTS AB AND SPORN MB. (1990). Structural and functional characterization of the transforming growth factor β3 promoter: a cAMP response element regulates basal and induced transcription. *J. Biol. Chem.*, **265**, 19128–19136.

LIPPMAN ME, DICKSON RB, GELMANN EP, ROSEN N, KNABBE C, BATES S, BRONZERT D, HUFF K AND KASID A. (1987). Growth regulation of human breast carcinoma occurs through regulated growth factor secretion. *J. Cell. Biochem.*, **35**, 1–16.

MACCALLUM J, BARTLETT JMS, THOMPSON AM, KEEN JC, DIXON JM AND MILLER WR. (1994). Expression of transforming growth factor beta mRNA isoforms in human breast cancer. *Br. J. Cancer*, **69**, 1006–1009.

MACCALLUM J, POULSOM R, HANBY AM AND MILLER WR. (1995). Expression and distribution of TGFβ mRNA isoforms in a small group of human breast cancers examined by *in situ* hybridisation. *The Breast*, **4**, 289–296.

MCCUNE BK, MULLIN BR, FLANDERS KC, JAFFURS WJ, MULLEN LT AND SPORN MB. (1992). Localization of transforming growth factor-β isotypes in lesions of the human breast. *Hum. Pathol.*, **23**, 13–20.

MANNING AM, WILLIAMS HC, GAME SM AND PARASKEVA C. (1991). Differential sensitivity of human colonic adenocarcinoma and carcinoma cells to transforming-growth factor-beta (tgf-beta) – conversion of an adenoma cell-line to a tumorigenic phenotype is accompanied by a reduced response to the inhibitor effects of tgf-beta. *Oncogene*, **6**, 1471–1476.

MIZUKAMI Y, TAJIRI K, NONOMURA A, NOGUCHI M, TANIYA T, KOYASAKI N, NAKAMURA S AND MATSUBARA F. (1991). Effects of tamoxifen, medroxyprogesterone acetate and estradiol on tumour growth and oncogene expression in MCF-7 breast cancer cell line transplanted into nude mice. *Anticancer Res.*, **11,** 1333–1338.

MOSES HL, TUCKER RF, LOEF EB, COFFEY, RJ JR, HALPER J AND SHIPLEY GD. (1985). Type beta transforming growth factor is a growth stimulator and a growth inhibitor. *Cancer Cells*, **3,** 65–71.

PEPPER MS, VASSALLI, J-D, ORCI L AND MONTESANO R. Biphasic effect of transforming growth factor-$\beta_1$ on *in vitro* angiogenesis. *Exp. Cell. Res.*, **204,** 356–363.

PIERCE DF, GORSKA AE, CHYTIL A, MEISE KS, PAGE DL, COFFEY RJ AND MOSES HL. (1995). Mammary tumour suppression by transforming growth factor β1 transgene expression. *Proc. Natl. Acad. Sci., USA*, **92,** 4254–4258.

ROBERTS AB, ANZANO MA, WAKEFIELD LM, ROCHE NS, STERN DF AND SPORN MB. (1985). Type β transforming growth factor: A bifunctional regulator of cellular growth. *Proc. Natl. Acad. Sci. USA*, **82,** 119–123.

SALOMON DS, CIARDIELLO F, VALVERIUS E, SAEKI T AND KIM N. (1989). Transforming growth factors in human breast cancer. *Biomed. Pharmacother.*, **43,** 661–667.

SCHULTZ GS AND GRANT MB. (1991). Neovascular growth factors. *Eye*, **5,** 178–180.

SPORN MB, ROBERTS AB, WAKEFIELD LM, GLICK AB AND DANIELPOUR D. (1990). Transforming growth factor-beta and suppression of carcinogenesis. In *Genetic Basis for Carcinogenesis: Tumour Suppression Genes and Oncogenes.* Knudson Jr AG, Stanbridge EJ, Sugimura T, Terada M and Watanbe S. (eds) pp. 259–266. Japan Science Society Press: Tokyo; and Taylor and Francis: London.

STEWART HJ. (1989). Clinical experience in the use of the anti-oestrogen tamoxifen in the treatment of breast cancer. *Proc. Royal. Soc. Edin.*, **95B,** 231–237.

THOMPSON AM, KERR DJ AND STEEL CM. (1991). Transforming growth factor β1 is implicated in the failure of tamoxifen therapy. *Br. J. Cancer*, **63,** 609–614.

TORRE-AMIONE G, BEAUCHAMP RD, KOEPPEN H, PARK BH, SCHREIBER H, MOSES HL AND ROWLEY DA. (1990). A highly immunogenic tumour transfected with a murine transforming growth factor type β1 cDNA escapes immune surveillance. *Proc. Natl. Acad. Sci.*, USA, 1490.

TRAVERS MYT, BARRETT-LEE PJ, BERGER U, LUQMANI YA, GAZET, J-C, POWLES TJ AND COOMBES RC. (1988). Growth factor expression in normal, benign and malignant breast tissue. *Br. Med. J.*, **296,** 1621–1624.

WALKER RA AND DEARING SJ. (1992). Transforming growth factor $beta_1$ in ductal carcinoma *in situ* and invasive carcinomas of the breast. *Eur. J. Cancer*, **28,** 641–644.

WALKER RA AND GALLACHER B. (1995). Determination of transforming growth factor $beta_1$ mRNA expression in breast carcinomas by *in situ* hybridization. *J. Pathol.*, **177,** 123–127.

WALKER RA, DEARING SJ AND GALLACHER B. (1994). Relationship of transforming growth factor β1 to extracellular matrix and stromal infiltrates in invasive breast carcinoma. *Br. J. Cancer*, **69,** 1160–1165.

WELCH DR, FABRA A AND NAKAJIMA M. (1990). Transforming growth factor β stimulates mammary adenocarcinoma cell invasion and metastatic potential. *Proc. Natl Acad. Sci. USA*, **87,** 7678–7682.